# Facile Synthesis of SnO_2_/LaFeO_3−X_N_X_ Composite: Photocatalytic Activity and Gas Sensing Performance

**DOI:** 10.3390/nano9081163

**Published:** 2019-08-14

**Authors:** Xi-Tao Yin, Davoud Dastan, Fa-Yu Wu, Jing Li

**Affiliations:** 1 The Key Laboratory of Chemical Metallurgy Engineering of Liaoning Province and School of Materials and Metallurgy, University of Science and Technology Liaoning, Anshan 114051, China; 2 Department of Materials Science and Engineering, Georgia Institute of Technology, Atlanta, GA 30332, USA

**Keywords:** LaFeO_3−X_N_X_, RhB degradation, charge separation, gas sensing

## Abstract

Here SnO_2_/LaFeO_3−X_N_X_ composite was fabricated using a wet chemical method and was applied to pollutants degradation and gas sensing for the first time. The composite exhibits high performance for photocatalytic degradation of Rhodamine B (RhB) dye and selectivity sensing of various gases. On the basis of the completed experiments, the improved RhB degradation and selective gas sensing performance resulted from the extended optical absorption via N_2_ incorporated surface states and enhanced charge separation via coupling SnO_2_. Using the scavengers trapping experiments, the superoxide radical (O_2_^•−^) was investigated as the major scavenger involved in the degradation of RhB over SnO_2_/LaFeO_3−X_N_X_ composite. In this paper, the probable reaction steps involved in the RhB dye degradation over SnO_2_/LaFeO_3−X_N_X_ composite are proposed. This work will provide reasonable strategies to fabricate LaFeO_3_-based proficient and stable catalysts for environmental purification. In addition, the result of the selectivity of gas performance is also presented.

## 1. Introduction

The degradation of various toxic, organic pollutants via semiconducting nanomaterials with the assistance of solar energy offers a hygienic and environmentally friendly method [1]. However, the detection and monitoring of highly toxic and flammable exhaust gases such as NO_2_, SO_2_, H_2_, and CO are vital for environmental remediation [2,3,4]. Semiconductor photocatalysts and gas sensors are widely employed for various pollutants oxidation and gas sensing in domestic and industrial surroundings because semiconductor metal oxides are ideal photocatalysts and gas sensors [5,6,7]. The design of low-cost, robust, and low-power-consuming semiconductor photocatalysts and gas sensors have attracted increasing attention due to their wide range of applications [8,9]. Research has focused on finding appropriate semiconducting nanomaterials with vital surface and bulk properties for high activity, sensitivity, and selectivity [10,11]. Among various metal oxide semiconductors, perovskite-type oxides have been given tremendous attention owing to their prospective applications in advanced technologies like chemical sensors, catalysts, solid oxide fuel cells, and magnetic materials [12]. The Lanthanum Ferrite (LaFeO_3_) perovskite (E_g_ = 2.0–2.8 eV) has been widely utilized in photocatalysis and gas sensing [13,14]. Yet, the performance of bare LaFeO_3_ is not sufficient for efficient photocatalysis and gas sensing owing to the short lifetime of photo-induced charge carriers [15]. To improve its performance, doping elements and heterojunctions with other semiconductors are widely used [16,17]. SnO_2_ is widely accepted as the best choice for gas sensing and pollutants degradation. Doping elements can greatly alter the surface states of nanomaterials, and coupling metal oxides can enhance charge separation thereby enhancing its performance for photocatalytic and gas sensing applications [18]. Thus, the unique characteristics of heterojunctions can improve the photocatalytic and gas sensing performance by promoting the charge carrier’s separation and transfer. For example, Zhang et al. [19] reported the synthesis of novel A- or B-site substituted LaFeO_3_/silica fiber (SF) composite photocatalysts that showed improved photocatalytic performance for degradation of Methylene blue (MB) dye under visible light irradiation. This enhanced activity was attributed to the synergistic effect caused by the doping which introduced surface states and the coupled silica fiber. Recently, Xu et al. [20] reported LaFeO_3_/g-C_3_N_4_ heterojunctions that showed enhanced activities for H_2_ evolution and MB degradation under visible light irradiation; these features were attributed to the remarkably enhanced charge carriers separation and transfer by the synergetic effect at the interface of the two components system. According to Armstrong et al. [21], LaFeO_3_ exhibits high adsorption capacity for NOx surface conversion in comparison with the LaMnO_3_. This difference was ascribed to the different B-site cations (Fe^3+^) in LaFeO_3_ when compared with the LaMnO_3_ with Mn^3+^ and Mn^4+^ valence states. Toan et al. [22] demonstrated that LaFeO_3_ films exhibit high sensitivity for sensing of different concentrations of CO, CH_4_, and NO_2_ gases in air atmosphere at various temperatures.

It is significant to explore the role of surface active species such as electron (e^−^), hole (h^+^), hydroxyl radical (•OH), and the superoxide radical (O_2_^•−^) in semiconductor photocatalysis and gas sensing. Primary oxidants (•OH) have no selectivity to organic pollutants. However, the O_2_^•−^ radical species generated in the conduction band of semiconductors by the interaction of electron with the adsorbed O_2_ can oxidize a variety of organic pollutants including dioxins, pesticides, and chlorinated pollutants. The mechanisms of pollutant degradation are strongly influenced by various catalysts and the model pollutants because the designed photocatalyst and the dominant active species decide the degradation mechanism [23,24]. However, the reaction mechanisms of pollutant degradation and gas sensing over a LaFeO_3_ semiconductor is not fully understood. Thus, it is necessary to develop a clear mechanism for pollutant degradation and gas sensing over a LaFeO_3_ semiconductor. To date, no works related to the fabrication of SnO_2_ coupled LaFeO_3−X_N_X_ catalyst for RhB dye degradation and gas sensing have been reported.

Here, we report the fabrication SnO_2_/LaFeO_3−X_N_X_ composite for high performance RhB dye degradation and gas sensing. On the basis of completed experiments, results indicate that the improved photocatalytic performance of SnO_2_/LaFeO_3−X_N_X_ for RhB dye degradation and gas sensing is accredited to the extended visible-light absorption via doping N_2_ and superior charge carrier’s separation via coupling SnO_2_. Further, it is confirmed by scavenger tapping results that O_2_^•−^ radical is the main oxidant species involved the degradation of RhB dye over SnO_2_/LaFeO_3−X_N_X_ composite. This work will trigger the development of LaFeO_3_-based high performance photocatalysts and gas sensors for environmental remediation and will help the scientific community to understand the surface chemistry of these processes.

## 2. Experimental Section

### 2.1. Synthesis of LaFeO_3_ Nanoparticles

LaFeO_3_ was fabricated via a sol–gel technique. In the given experiment, equimolar amount (i.e., 0.04 mol) of La(NO_3_)·6H_2_O and Fe(NO_3_)_3_·9H_2_O was dissolved into 25 mL mixture of ethylene glycol (EG) and water (1:1). The solution was magnetically stirred at room temperature for 4 h to obtain a gel. The gel was dried in oven at 100 °C overnight. Finally, the dry powder was kept in a ceramic crucible and annealed at 600 °C (heating rate = 5 °C min^−1^) in a muffle furnace for 2 h to obtain LaFeO_3_. All reagents were purchased from Aladdin Company. (Aladdin Reagent (Shanghai) Co., Ltd., Shanghai, China).

### 2.2. Synthesis of LaFeO_3−X_N_X_ Oxynitride

To prepare LaFeO_3−X_N_X_ oxynitride, 2 g of the fabricated LaFeO_3_ was taken in a ceramic boat and kept in a quartz reactor. Next, the quartz reactor was carefully sealed and N_2_ gas was allowed to pass through a saturated system concentrated with aqueous ammonia solution. The N_2_ was used as a carrier gas for transportation of ammonia vapor into the reactor. The ammonolysis reaction was carried out at 500 °C for 6 h. When room temperature attained, the LaFeO_3−X_N_X_ oxynitride photocatalyst was collected.

### 2.3. Fabrication of SnO_2_/LaFeO_3−X_N_X_ Nanocomposite

To fabricate SnO_2_ coupled LaFeO_3−X_N_X_ oxynitride composite (SnO_2_/LaFeO_3−X_N_X_), 1 g of LaFeO_3−X_N_X_ oxynitride catalyst was dispersed into a 50 mL of ethanol and water mixture (1:1). Subsequently, 5% by mass of SnO_2_ was added to it. After continuous stirring for 6 h, the mixture was dried in an oven at 80 °C overnight. The powder was finally annealed at 450 °C (N_2_ atmosphere) for 2 h to obtain SnO_2_/LaFeO_3−X_N_X_ composite.

### 2.4. Characterization

The crystalline morphology of the photocatalysts was examined by an X-ray diffractometer (D8-Advance, Bruker Inc., Karlsruhe, Germany). The UV-visible, diffuse reflectance spectroscopy spectra (UV-vis DRS) were obtained with a Perkin Elmer UV/Vis spectrometer (Lambda-35, PerkinElmer Inc.). The scanning electron microscopy (SEM) micrographs and the energy dispersive spectroscopy (EDS) spectra were obtained with a scanning electron microscope (Geminisem-300-7112, Carl Zeiss Inc., Dresden, Germany). The elemental chemical composition was examined by the X-ray photoelectron spectrometer (AXIS-ULTRA DLD-600W, Shimadzu Inc., Kyoto, Japan). The photoluminescence (PL) and fluorescence (FL) spectra were obtained with a spectrofluoro-photometer (FP-6500, Spectroscopic Co. Ltd., Kyoto, Japan) at 325 nm excitation wavelength.

### 2.5. Photoelectrochemical Measurements

For photoelectrochemical (PEC) experiments, the electrode films were prepared in advance by a doctor-blade procedure using a high temperature 3 m 2380 scotch tape as the spacer. In a typical procedure, 25 mg of each photocatalyst was dispersed in 1 mL of isopropyl alcohol and kept under ultrasonic treatment for 30 min followed by magnetic stirring for 30 min. Then, 25 mg of highly grinded Macrogol-6000 was added to the photocatalyst solution and the ultrasonic treatment was repeated with magnetic stirring for 30 min each. After that, acetylacetone (0.05 mL) was drop wise added to the solution and vigorously stirred for 48 h. Finally, the product was pasted on thoroughly washed, conductive, fluorine-doped, tin-oxide (FTO) glass substrates (10–15 ohm/sq, Xinyan Tech Ltd., Chengdu, China) and sintered at 450 °C (5 °C min^−1^) for 30 min. The FTO glass substrates were then cut in to 1.0 cm × 3.0 cm pieces containing the film surface area of 1 cm × 1 cm.

### 2.6. Photoactivity Evaluation

The experiments were performed in a 100 mL volume glass beaker. A 150 W Xe-lamp (GYZ220, CEAULIGHT. Inc., Beijing, China) with cut-off filter (420 nm) was used as the irradiation source. During photocatalytic process, 50 mg of the photocatalyst was taken into 40 mL of RhB-dye solution (10 mg/L) and continuously stirred for 10 min to reach the adsorption equilibrium. The solution was kept under visible-light irradiation for 2 h. After regular intervals of 30 min, an appropriate amount of RhB-dye solution was taken with a syringe and centrifuged to measure its concentration with a Perkin Elmer UV/Vis spectrometer (Lambda-35, PerkinElmer Inc.), at characteristic optical absorption of 553 nm.

### 2.7. Gas Sensing Evaluation

Sample preparation: A Pt interdigital electrode was coated on the alumina substrate by screen printing. After annealing at 800 °C, the Pt interdigitated electrode was firmly adhered to the alumina substrate. The photocatalyst powder was mixed with a small amount of organic binder. The binder was prepared by stirring terpineol and ethyl cellulose in a ratio of 1 g of ethyl cellulose to 10 mL of terpineol. Slurry of photocatalyst for preparing a thick film was obtained. The slurry was uniformly coated on an alumina ceramic sheet having a Pt electrode by a screen printing method to prepare a thick film, and the obtained thick film sheet was annealed at 550 °C for 3 h.

Gas Sensitivity Test: The test system consists of a multi-stage gas dilution system, an electric resistance furnace, and an electrochemical workstation (CHI660E, CH Instruments Inc, Shanghai, China). The sample was placed in an electric resistance furnace connected to two electrodes and an external gas sensing recorder. The experiments were performed at 400 °C. Three kinds of gases, H_2_, CO, and SO_2_, were chosen, and their concentration in air with exposure time was 500 mL/min. The test gas flow rate was 10 mL/min, and the exposure time was 300–600 s. The test system uses an electrochemical workstation with a set voltage of 4 V. To obtain the “I-t” curves of the material, the current-time relationship was used. To obtain an “R-t” curve, the resistance was calculated using Ohm’s law.

## 3. Results and Discussion

### 3.1. Structural Morphology and Chemical Composition

**Figure 1:**
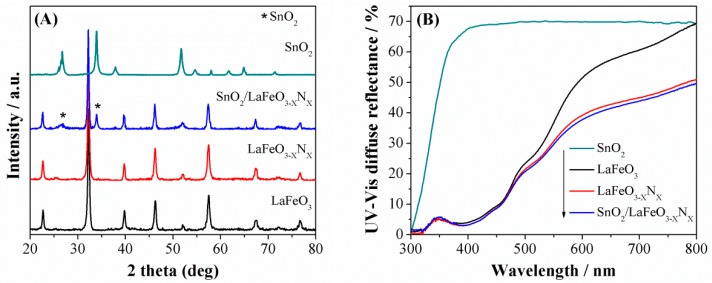
XRD patterns (**A**) and UV-Visible diffuse reflectance spectra (**B**) of LaFeO_3_, LaFeO_3−X_N_X_, SnO_2_/LaFeO_3−X_N_X_, and SnO_2_ photocatalysts.

**Figure 2:**
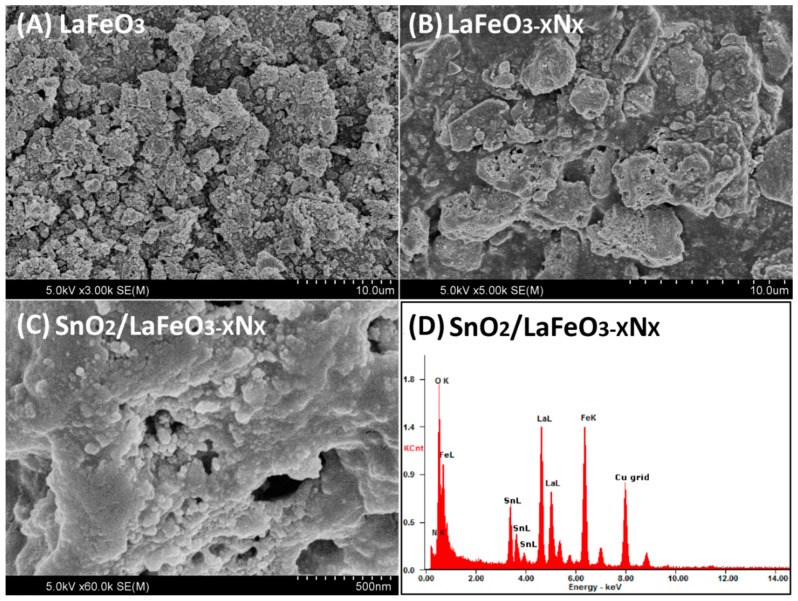
Scanning electron microscopy (SEM) images (**A**–**C**) of LaFeO_3_, LaFeO_3−X_N_X_, and SnO_2_/LaFeO_3−X_N_X_ photocatalysts, and Energy Dispersive X-ray (EDX) spectrum (**D**) of SnO_2_/LaFeO_3−X_N_X_ photocatalyst.

**Figure 3:**
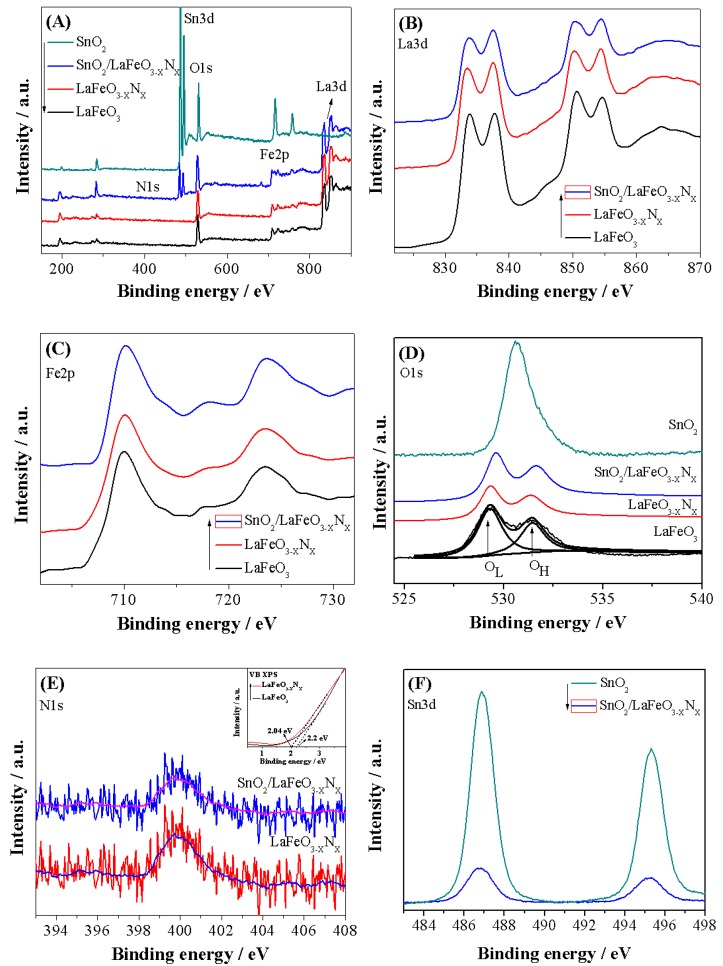
X-ray photoelectron spectroscopy (XPS) survey spectra (**A**) of LaFeO_3_, LaFeO_3−X_N_X_, SnO_2_, and SnO_2_/LaFeO_3−X_N_X_ photocatalysts, high resolution La3d XPS spectra (**B**) and Fe2p XPS spectra (**C**) of LaFeO_3_, LaFeO_3−X_N_X_, and SnO_2_/LaFeO_3−X_N_X_ photocatalysts, O1s XPS spectra (**D**) of LaFeO_3_, LaFeO_3−X_N_X_, SnO_2_, and SnO_2_/LaFeO_3−X_N_X_ photocatalysts, N1s XPS spectra with inset valence band XPS spectra of LaFeO_3_ and LaFeO_3−X_N_X_ (**E**) and Sn3d XPS spectra (**F**) of LaFeO_3−X_N_X_ and SnO_2_/LaFeO_3−X_N_X_ photocatalysts.

The X-ray diffraction (XRD) patterns of the photocatalysts are shown in Figure 1A. The XRD patterns of LaFeO_3_ can be assigned to the highly crystalline perovskite-type orthorhombic phase and indexed to the JCPDS No. 37-1493 [25]. The crystallite size were calculated at intense diffraction peak located at 32.19° (hkl = 121) using Debye Scherrer equation (D_size_ = Kλ/βCosθ) [26]. The calculated crystallite size of LaFeO_3_, LaFeO_3−X_N_X_, and SnO_2_/LaFeO_3−X_N_X_ were 100, 107, and 108 nm, respectively. Notably, the intense XRD signal of LaFeO_3−X_N_X_ is slightly red shifted which is attributable to the partial substitution of oxygen anion (O^2−^, radius = 1.4 Å) with that of the nitrogen ones (N^3−^, radius = 1.5 Å). Accordingly, O^2−^ has higher electro-negativity (3.5) than N^3−^ (3.07). Thus the robust covalent metal-N_2_ bonding reduced the energy band gap of LaFeO_3_ and extended its optical absorption behavior [27]. When SnO_2_ was coupled with LaFeO_3−X_N_X_, the peaks related to SnO_2_ also appeared in the composite, suggesting the successful formation of heterojunction. In addition, SnO_2_ coupling did not affect the crystalline phase morphology of LaFeO_3_. The UV-Visible diffuse reflectance spectra (UV-Vis DRS) were measured to investigate the absorption behavior of the photocatalysts, as shown in Figure 1B. The energy band gaps of LaFeO_3_ and LaFeO_3−X_N_X_ photocatalysts were estimated to be 2.0 and 1.84 eV, respectively, based on the energy band gap equation E_g_ = 1240/λ. Further, the SnO_2_ coupling did not affect the energy band gap of LaFeO_3−X_N_X_. The scanning electron microscopy (SEM) micrographs were obtained to examine the morphology of the photocatalysts. Figure 2A reveals that LaFeO_3_ nanoparticles have an average crystallite size of about 100 nm and is in accordance with the XRD crystallite size. As seen in Figure 2B, after ammonolysis reaction for 6 h, the morphology of LaFeO_3_ was slightly changed and its particle size was enlarged. The SEM micrograph of SnO_2_/LaFeO_3−X_N_X_ composite, as seen in Figure 2C, shows that the nano-size SnO_2_ particles are present on the surface of LaFeO_3_ photocatalyst. The energy dispersive spectroscopy (EDS) spectrum of SnO_2_/LaFeO_3−X_N_X_ composite, as seen in Figure 2D, shows La, Fe, O, N, and Sn elements, which demonstrate that N was successfully integrated into the crystal lattice of LaFeO_3,_ and SnO_2_ was coupled. The X-ray photoelectron spectroscopy (XPS) spectra were obtained to investigate the chemical composition of the photocatalysts. The binding energies were standardized for specimen charging using C1s as the reference (peak position = 285.0 eV) [28]. The XPS survey spectra of LaFeO_3_, LaFeO_3−X_N_X_, and SnO_2_/LaFeO_3−X_N_X_ are shown in Figure 3A. From the high resolution XPS of La3d seen in Figure 3B, the binding energy peaks at 833.5 and 850.4 eV, respectively, corresponds to the La3d_5/2_ and La3d_3/2_ energy levels. Notably, both of the peaks exhibit a satellite peak at a distance of 4.0 eV and the spin-orbital splitting of La3d_5/2_ and La3d_3/2_ is about 16.9 eV. This reveals that La3d exists in +3 oxidation state. As seen in Figure 3C, the high resolution XPS spectra of Fe2p reveals the presence of Fe2p_3/2_ and Fe2p_1/2_ energy levels at 710 and 723.5 eV, respectively. This confirms the existence of Fe2p in +3 oxidation state [29]. As seen in Figure 3D, the high resolution XPS spectra of O1s shows binding energy peaks at 529.5 and 531.5 eV, respectively, for lattice oxygen (OL, La–O, and Fe–O bonds), and hydroxyl (OH) one [30]. As seen in Figure 3E, the high resolution N1s XPS spectra for LaFeO_3−X_N_X_, and SnO_2_/LaFeO_3−X_N_X_ photocatalysts are broad and sited at binding energy of ~399 eV [31,32]. As seen in Figure 3F, the high resolution Sn3d XPS spectra display peaks at binding energies of 486.9 and 495.2 eV, respectively, that correspond to the Sn3d_5/2_ and Sn3d_3/2_ energy levels in pure SnO_2_ and SnO_2_/LaFeO_3−X_N_X_ photocatalysts [33]. Thus, it is confirmed that N is successfully integrated into the matrix of LaFeO_3_ and SnO_2_ is coupled to it.

### 3.2. Photo-Induced Charge Properties

**Figure 4:**
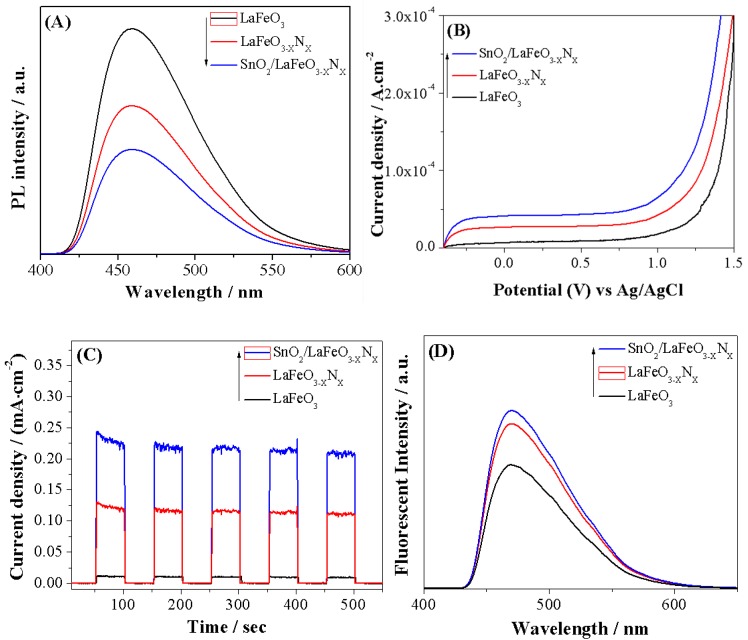
Photoluminescence (PL) spectra (**A**), photoelectrochemical (PEC) I-V curves (**B**), PEC I-t curves (**C**) and fluorescence (FL) spectra (**D**) of LaFeO_3_, LaFeO_3−X_N_X_, and SnO_2_/LaFeO_3−X_N_X_ photocatalysts.

To inspect the photo-induced charge properties of LaFeO_3_, LaFeO_3−X_N_X_, and SnO_2_/LaFeO_3−X_N_X_ photocatalysts, photoluminescence (PL) spectra were measured. The PL technique is highly sensitive and is used to explore the surface active sites of semiconductor nanomaterials. From PL spectroscopy, we can obtain knowledge regarding defects in semiconductor surfaces, oxygen vacancies, trapping, immigration, and transfer of charges, [34]. As seen in Figure 4A, LaFeO_3_ exhibits a strong PL response, which is related to the fast recombination rate of photo-generated charges. Notably, the PL intensity peak of LaFeO_3−X_N_X_ is remarkably decreased and further decreased after coupling SnO_2_. This demonstrates that the charge carrier’s recombination in LaFeO_3−X_N_X_ is considerably reduced. To further investigate the enhanced charge carrier’s separation, photoelectrochemical (PEC) I-V and I-t curves were measured, as shown in Figure 4B,C. LaFeO_3_ exhibits a low photo-current density signal. Notably, the photo-current density signal of LaFeO_3−X_N_X_ photocatalyst is obviously improved and that of the SnO_2_/LaFeO_3−X_N_X_ photocatalyst is significant. The •OH radical can also provide information about the photo-induced charge carrier’s separation during semiconductor photocatalysis [35]. The strong fluorescence (FL) signal reflects a large amount of the •OH radical and high charge carrier’s separation. Thus, we have detected the •OH amount by the coumarin fluorescent method. In this method, the coumarin react with •OH radical and produce 7-hydroxy-coumarin luminescent species. As shown in Figure 4D, LaFeO_3_ photocatalyst produced small amount of •OH radical. However, the amount of •OH radical produced over LaFeO_3−X_N_X_ is remarkably increased and significant for the SnO_2_/LaFeO_3−X_N_X_ nanocomposite. This is in agreement with the PL and PEC results, signifying that charge carrier’s separation is remarkably improved.

### 3.3. Photocatalytic and Gas Sensing Performance

**Figure 5:**
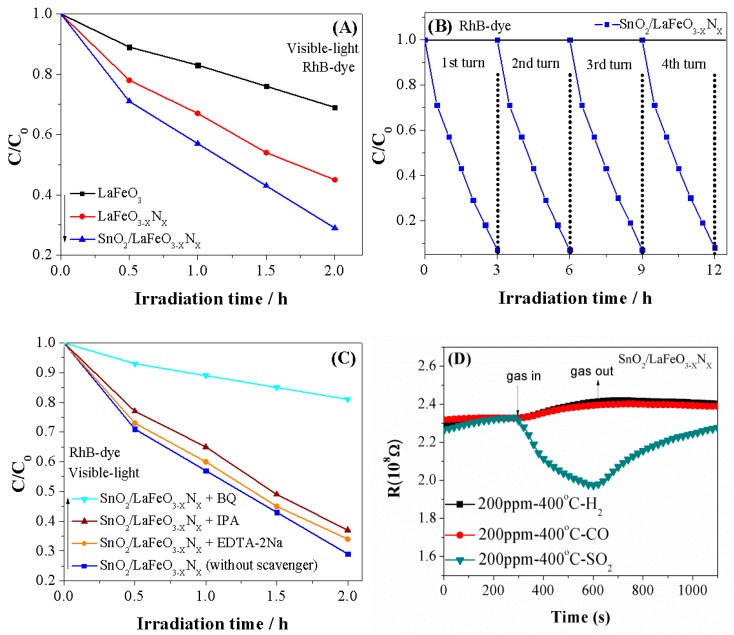
Photocatalytic activities for RhB-dye degradation (**A**) of LaFeO_3_, LaFeO_3−X_N_X_ and SnO_2_/LaFeO_3−X_N_X_ photocatalysts. Photostability and recyclability test (**B**) and scavengers trapping experiments for RhB dye degradation (**C**) of SnO_2_/LaFeO_3−X_N_X_ photocatalyst. Gas sensing performance of SnO_2_/LaFeO_3−X_N_X_ photocatalyst for 200 ppm of H_2_, CO, and SO_2_ gases at 400 °C (**D**).

The visible-light activity of the photocatalysts was performed for the RhB-dye degradation and the results are provided in Figure 5A. Notably, LaFeO_3_ photocatalyst degraded only 31% of RhB-dye beneath visible-light irradiation for 2 h. The degradation of RhB-dye over LaFeO_3−X_N_X_ photocatalyst is considerably high (i.e., 55%). Interestingly, the degradation of RhB-dye over SnO_2_/LaFeO_3−X_N_X_ composite is significant (i.e., 71%), higher than reported thus far under the same experimental conditions as depicted in Table 1. Notably, the improved photocatalytic activity of LaFeO_3_ photocatalyst for RhB-dye degradation is attributed to the extended optical absorption via doping N and to the enhanced charge carrier’s separation via coupling SnO_2_. Thus, the designed SnO_2_/LaFeO_3−X_N_X_ nanocomposite is favorable in terms of the degradation of organic dye pollutants in comparison with the LaFeO_3_ and LaFeO_3−X_N_X_ photocatalysts. To check the stability of SnO_2_/LaFeO_3−X_N_X_ photocatalyst for RhB-dye degradation, we carried out photocatalytic stability and recyclability tests under visible-light irradiation for four consecutive recycles (each lasting 3 h). As seen in Figure 5B, the photocatalyst does not show any apparent loss in the activity even after a 12-hour irradiation period. This demonstrates that the SnO_2_/LaFeO_3−X_N_X_ photocatalyst is very stable and does not photocorrode during the photocatalytic reaction. To confirm which radical species is involved in the reaction process, we carried out scavengers trapping experiments in the presence of Isopropyl-alcohol (IPA, •OH scavenger), Benzo-quinone (BQ, O_2_^•−^ scavenger), and EDTA-2Na (h^+^, scavenger) [36]. Appropriate amounts of each scavenger (1 mM stock) was added to the RhB-dye solution before adding the catalyst and stirred for 15 min. After catalyst addition, the sample was illuminated under visible light for 2 h. Figure 5C shows that the degradation of RhB-dye over SnO_2_/LaFeO_3−X_N_X_ photocatalyst is slightly suppressed in the presence of EDTA-2Na and IPA scavengers while sturdily inhibited in the presence of BQ scavenger. This reveals that the O_2_^•−^ radical is the predominant active species involved in the degradation of RhB-dye over SnO_2_/LaFeO_3−X_N_X_ photocatalyst, and the role of h^+^ and •OH is less significant.

For the gas sensing activity, the sensing mechanism of n-type metal oxide semiconductors is normally associated with the space charge region formation on the oxide surfaces, owing to the electron capture on the adsorbed oxygen species [37,38]. Whenever a sensor detects a reactive gas, the gas reacts with the surface oxygen species to release electrons, which are then trapped by the adsorbed oxygen species. As a result, the conductivity of metal oxide increases. The mechanism for p-type metal oxide semiconductors is opposite. Thus, on the basis of the sensing mechanism, the main disadvantage of this type of sensor is its poor selectivity to various gases having the similar (reducing or oxidizing) nature [39,40]. However, the SnO_2_/LaFeO_3−X_N_X_ material has different sensing behaviors to the homogeneous gases CO, H_2_, and SO_2_, as shown in Figure 5D. The resistance of the sensor material will increase when detecting CO and H_2_, but decrease when detecting SO_2_. SO_2_ commonly presents similar gas sensing behavior with CO and H_2_ when adsorbed on the surface of metal oxide sensing materials. However, it presented an opposite behavior here, which may contribute to the corrosive effect of SO_2_. The SO_2_ adsorbed on the surface of the sensing materials to form the adsorption species SO_3_ [41], which may cause a change of composition of the sensing material surface. Thus, the sensing behavior and type conductivity may transfer from p-type to n-type dominated. This indicates that the SnO_2_/LaFeO_3−X_N_X_ material may be a promising candidate for applications in solving the poor selectivity of these types of sensors to SO_2_.

### 3.4. Discussion

**Figure 6:**
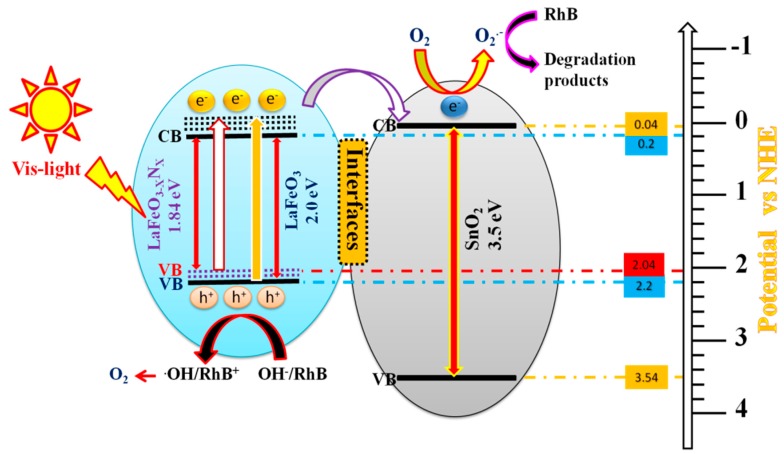
Schematic of the energy band gaps, charge carrier’s transfer and separation, and the photocatalytic processes over SnO_2_/LaFeO_3−X_N_X_ photocatalyst under visible-light irradiation.

According to the above results, it has been demonstrated that the activity of LaFeO_3_ photocatalyst for RhB-dye degradation can be greatly improved by doping N and then coupling SnO_2_. This is a result of the newly formed surface states near the valence band top that effectively traps the photogenerated holes and allows the energetic electrons to transfer to the conduction band of SnO_2_. To understand the charge transfer mechanism in the fabricated SnO_2_/LaFeO_3−X_N_X_ composite, we proposed an energy level diagram, as shown in Figure 6. As confirmed by the UV-Visible DRS spectra, the energy band gap of LaFeO_3_ photocatalyst is 2.0 eV, corresponding to the light absorption of λ ≤ 620 nm according to the energy band gap equation E_g_ = 1240/λ where “E_g_” is the energy band gap (eV) and “λ” is the wavelength (nm) of absorbed photons. From the valence band XPS spectra, as seen in Figure 3E inset, the valence band level of LaFeO_3_ photocatalyst stands at 2.2 eV. After doping N, its band gap is reduced to 1.84 eV through a shift of the valence band upward to 2.04 eV which corresponds to the light absorption of approximately λ ≤ 674 nm. On the other hand, the energy band gap of SnO_2_ is 3.5 eV with its valence and conduction bands located at 3.54 and 0.04 eV, respectively. Thus, SnO_2_ can only be excited under UV-light (λ ≤ 354 nm). It is recommended that the photogenerated electrons of LaFeO_3_ and LaFeO_3−X_N_X_ could be relaxed to their conduction band bottom in a small time scale with energy loss, resulting in the poor charge carrier’s separation and low photocatalytic performance. However, when a heterojunction is formed between SnO_2_ and LaFeO_3−X_N_X_ photocatalysts and the composite is excited under visible-light, the generated highly energetic electrons of LaFeO_3−X_N_X_ would transfer to the conduction band of SnO_2_, leaving the excited holes in its valence band. The electrons in the conduction band of SnO_2_ would probably react with the surface-adsorbed O_2_ to generate superoxide radicals (O_2_^•−^) that will continue the oxidation of RhB-dye pollutant. On the other hand, the holes left in the valence band of LaFeO_3−X_N_X_ would react with the surface adsorbed water and/or OH^−^ species to produce •OH radicals and then to evolve O_2_. Thus, the produced active intermediates would take part in the organic pollutants degradation. In this way, the photogenerated charge carrier’s separation will significantly improve. Via this phenomenon, it is simple to make the photocatalytic activity of composite photocatalysts more practical. The fabricated visible-light-responsive nanocomposite can be utilized for effective environmental remediation. The probable stepwise reactions involved in the photocatalytic degradation of RhB over SnO_2_/LaFeO_3−X_N_X_ composite are described in Equations (1) to (5).

SnO_2_/LaFeO_3−X_N_X_ + *h*ν → SnO_2_ (No e^−^/h^+^ pairs)/LaFeO_3−X_N_X_ (e^−^ + h^+^) (1)

SnO_2_ (No e^−^/h^+^ pairs)/LaFeO_3−X_N_X_ (e^−^ + h^+^) → SnO_2_ (e^−^)/LaFeO_3−X_N_X_ (h^+^) (2)

SnO_2_ (e^−^ + O_2_) →O_2_•^−^ (3)

SnO_2_ (O_2_•^−^ + RhB) → degradation products (4)

LaFeO_3−X_N_X_ (h^+^ + RhB) → degradation products (5)

## 4. Conclusions

SnO_2_/LaFeO_3−X_N_X_ nanocomposite was fabricated via a wet chemical route that exhibited superior visible-light activity for RhB-dye degradation in comparison with the LaFeO_3−X_N_X_ and LaFeO_3_ photocatalysts. This is attributable to the extended optical absorption and enhanced photo-induced charge carrier’s separation via N-introduced surface states and the coupled SnO_2_. This work will trigger reasonable routes for the fabrication of highly efficient visible-light photocatalysts for effective environmental remediation. In addition, the SnO_2_/LaFeO_3−X_N_X_ material would be a promising candidate for applications in solving the poor selectivity of this type of sensors.

## Figures and Tables

**Table 1:** Comparison of the RhB degradation activity of our photocatalyst with previous reports.

S. #	Photocatalyst	Light Source	RhB Degradation (%)	References
1	β-FeSe/g-C_3_N_4_	300 W Xe-lamp	45 % (3 h)	[42]
2	R40-BiFeO_3−X_	300 W Xe-lamp	60 % (6 h)	[43]
3	g-C_3_N_4_/ SAPO-5	300 W Xe-lamp	47.15 % (2.5 h)	[44]
4	Bi_2_Ti_2_O_7_/TiO_2_	150 W Xe-lamp	90 % (6 h)	[45]
5	SnO_2_/LaFeO_3−X_N_X_	300 W Xe-lamp	71 % (2 h)	This work
